# Azole-Driven Cross-Resistance and Transporter Gene Expression in *Malassezia* Yeasts

**DOI:** 10.3390/microorganisms14061315

**Published:** 2026-06-12

**Authors:** Ying Zhou Soo, Shi Mun Lee, Thomas L. Dawson, Cheryl Leong

**Affiliations:** 1 A*STAR Skin Research Labs (A*SRL), Agency for Science, Technology and Research (A*STAR) & Skin Research Institute of Singapore (SRIS), 11 Mandalay Rd, #17-01, Singapore 308232, Singapore; 2 Center for Cell Death, Injury & Regeneration, Department of Drug Discovery & Biomedical Sciences, Medical University of South Carolina, Charleston, SC 29401, USA

**Keywords:** antifungal, cross-resistance, azoles, *Malassezia*, transporters

## Abstract

*Malassezia* are commensal lipid dependent yeasts which can cause opportunistic skin infection. Topical imidazole antifungals such as clotrimazole and ketoconazole are the frontline treatment. However, the tendency of fungal infections to recur, combined with the emergence of multi-azole-resistant *Malassezia* isolates means that many patients have used these antifungal treatments repeatedly or for extended durations with limited efficacy. While the impact of single azole treatments has been studied, the ability of specific azoles to induce cross-resistance is unclear. Understanding the effect of prior exposure of one treatment on susceptibility to other antifungals is important in the selection of the appropriate treatment to avoid driving the evolution of greater resistance. We previously identified drug transporters from the ATP-Binding Cassette (ABC) and Major Facilitator Superfamily (MFS) to be upregulated on extended exposure to clotrimazole. In this study, we investigated the effect of extended clotrimazole, ketoconazole and fluconazole exposure on antifungal cross-resistance profiles and examined the expression of the MFS transporters OPT1 and FLR1 in resistance emergence. We observed that treatment with clotrimazole was associated with increased cross-resistance to other antifungals. Ketoconazole treatment caused elevated MICs in all tested antifungals that did not decrease after drug removal. These findings advance our understanding of fungal adaptive resistance mechanisms and inform improved antifungal strategies to mitigate resistance development.

## 1. Introduction

The *Malassezia* genus comprises 19 species to date [1,2], with up to eight species observed to be anthrophilic [3]. These lipid-dependent yeast species are isolated specifically from mammalian skin, although there is evidence of *Malassezia* being detected in the gut and central nervous system [4]. *M. globosa*, *M. restricta*, *M. furfur* and *M. sympodialis* are the most well described mycobiome commensals and are commonly found on healthy human skin [5]. In conditions of microbiome dysbiosis, they are also associated with cutaneous infections such as dandruff, seborrheic dermatitis and pityriasis versicolor [6]. Systemic infection is rare but has been reported in immunocompromised individuals [7]. Outbreaks of nosocomial infections in neonatal wards have been linked to both animal-to-human transmission [8,9] and enteral tube feeding, in which *Malassezia* utilize the lipids in milk to colonize catheter tubing, thereby entering the bloodstream and leading to systemic infection [10].

Azole antifungals are the primary standard-of-care treatment for fungal infections [11]. The imidazoles clotrimazole, miconazole and ketoconazole are used mainly in topical skin care products such as creams and shampoos, whereas triazoles such as fluconazole and itraconazole are more widely used to treat systemic infection [12]. Azole-resistant isolates of *Malassezia* are a problem as the genus is intrinsically resistant to the echinocandin drug class, leaving few treatment alternatives [13]. Clusters of *M. furfur* isolates have been previously identified to have reduced susceptibility to fluconazole, voriconazole, clotrimazole and miconazole [14]. Reduced susceptibility primarily to ketoconazole has also been described for commensal and clinical isolates of *M. restricta*. This has been attributed to (1) genomic multiplication of genes associated with ergosterol synthesis, mitochondrial iron metabolism, and oxidative stress response or (2) the overexpression of drug efflux pumps such as PDR10 and ATM1 [14,15,16].

Azole cross-resistance can be defined as the event in which exposure to one azole rapidly confers a resistant phenotype to other azole drugs, likely because of a shared target of mechanism of action [17]. Existing studies have evaluated the impact of the use of a single azole treatment on the susceptibility of a strain to other azoles [18]. However, it is not clear whether the above-mentioned mechanisms remain conserved across all azoles or whether one azole is more likely to evoke cross-resistance above another. Azole cross-resistance has been reported in clinical isolates of *Candida* [19] and *M. pachydermatis* [20], but antifungal susceptibility profiling alone does not enable us to identify the primary mechanism or trigger for the organisms to invoke their resistant phenotypes.

There are two major families of multidrug transporters [21]. ATP-Binding Cassette (ABC) transporters are primary transporters involved in active cellular drug transport. To date, most major transporters described to play a role in drug resistance belong to this family (e.g., P-glycoprotein (ABCB1) and breast cancer resistance protein (ABCG2)) [22]. The Major Facilitator Superfamily (MFS) is the largest group of secondary transporters involved in the cellular transport of carbohydrates, drugs, metabolites, neurotransmitters, nucleosides, amino acids, peptides, organic and inorganic anions, cations and various Kreb’s cycle intermediates, and they function as uniporters, symporters or antiporters [23]. There is increasing evidence that these drug transporters are involved in multidrug resistance [18,23].

The *M. furfur* strain, CBS 14141, is representative of a pathogenic strain isolated from the blood during systemic infection, that has evolved intrinsic resistance to several azoles. This strain exhibits constitutively high multidrug transporter expression along with mutations in the lanosterol 14-alpha-demethylase gene, CYP51A1, which is responsible for ergosterol synthesis in the fungal cell wall [14]. A CRISPR–Cas9-mediated *PDR10* knockout in this strain displayed reduced azole susceptibility relative to its wild-type parent strain, implicating *PDR10* as an important determinant of the resistant phenotype [14,24]. In the susceptible CBS 7982 *M. furfur* strain, prolonged clotrimazole exposure has been shown to induce elevated MICs, accompanied by upregulation of multidrug transporter genes, including *PDR10* [14].

In this study, we extend previous work by examining how exposure of the susceptible *M. furfur* strain CBS 7982 to clotrimazole, ketoconazole, or fluconazole reshapes its susceptibility profile to other azoles, and by assessing the contribution of the secondary MFS transporters OPT1 and FLR1 to the emergence of a transient adaptive phenotype. We also investigate whether these transporters facilitate short-lived, drug-induced tolerance by evaluating their recovery after drug withdrawal. A clearer understanding of how individual azoles differentially modulate cross-resistance, together with insight into the underlying transport-mediated mechanisms, will be critical for optimizing antifungal selection and designing treatment strategies that minimize the development and spread of resistance.

## 2. Materials and Methods

### 2.1. Malassezia Strains and Culture Conditions

*Malassezia* furfur strains CBS 7982 and CBS 14141 were obtained from the Westerdijik Fungal Diversity Institute. Cells were cultured in modified mDixon broth comprising (per 1 L) 20 g of dessicated Oxbile (B3883, Sigma Aldrich, Singapore), 6 g of peptone (BD 211677, Becton Dickinson, Singapore), 36 g of malt extract (70167, Sigma-Aldrich, Singapore), 2 mL of oleic acid (27728, Sigma Aldrich, Singapore), 10 mL of Tween 40 (P1504, Sigma Aldrich, Singapore) and 4 mL of 50% glycerol (BUG1120, 1st Base, Singapore) at 32 °C and maintained every 2–3 days.

For single extended treatment conditions, fresh cultures of CBS 7982 were set up in triplicate and treated with 8 μg/mL of clotrimazole or 2 μg/mL of fluconazole or 0.12 μg/mL for four weeks, with media and drug replaced on a weekly basis. We elected to use MICs two to four steps higher than the measured MIC to establish a high-stress, sublethal condition representative of localized “high-dose” environments found in topical applications and biofilm matrices. The specific doses were adjusted based on the maximum dose the strain could tolerate without compromising live cell load required for downstream broth microdilution assays and gene expression analysis. The drug treatment was removed from the media on weeks five and six. An aliquot of cell suspension was taken on a weekly basis to perform antifungal susceptibility profiling, and RNA extraction was performed for cell pellets obtained at Weeks 0, 3, 4 and 6 for gene expression analysis.

### 2.2. Broth Microdilution for Antifungal Susceptibility Testing

A broth microdilution assay was used to determine antifungal susceptibility testing and was performed as described by Leong et al. [13]. The antifungals, amphotericin B, terbinafine, clotrimazole, miconazole, itraconazole, fluconazole, voriconazole, and ketoconazole were purchased from Sigma-Aldrich, Singapore. Drug dilutions were performed using 200× stock solutions in fresh OptiMAL medium [13], containing 12.5 µg/mL of resazurin sodium salt in accordance with CLSI and EUCAST guidelines. Fresh cultures of *Malassezia* were used for each assay. To achieve a final cell density of 5 × 10^3^ to 5 × 10^4^ CFU/mL, a 50 μL yeast inoculum was added to 50 μL of 2× antifungal solution in OptiMAL. For CFU counting, 10 μL of yeast inoculum diluted 10 times was plated onto a an mDixon agar plate and incubated for 4 to 7 days at 35 °C (expect 10 to 100 colonies per plate). Plates were read within 24 to 48 h to preparation (when positive control turns pink) and each assay was performed in triplicate plates.

### 2.3. Data Analysis

Changes in MIC during drug exposure (Weeks 1–4) were expressed as log2 fold changes relative to the baseline MIC at Week 0 for each antifungal treatment group. Changes in MIC following drug withdrawal (Weeks 5–6) were expressed as log2 fold changes relative to the Week 4 MIC for the corresponding treatment group. Associations between MIC changes in treated cultures and those in the respective control treatment group (clotrimazole, ketoconazole, or fluconazole) were assessed using Spearman’s rank correlation with the 95% confidence interval, one-tailed test to evaluate positive correlations to treatment group. For group-wise comparisons, MIC data were analyzed using a two-way ANOVA followed by Tukey’s multiple comparisons test.

### 2.4. Rhodamine 6G Efflux Assay

Briefly, cells were treated with the respective concentrations of ketoconazole, clotrimazole and fluconazole at 32 °C. After 1 week, cells were pelleted and washed 3× in PBS followed by incubation with 10 µM of Rhodamine 6G in PBS (glucose starvation) for 30 min. This was followed by washing twice in ice-cold PBS. Lastly, cells were resuspended in PBS with 2% glucose and incubated at 32 °C; cell aliquots were harvested at 0, 5, 15 and 30 min intervals; and the supernatants were collected for measurement on a fluorescence plate reader (excitation/emission: 525 nm/550 nm). Efflux activity was measured as a fold change over the fluorescence signal detected at T_0_.

### 2.5. RNA Extraction

*Malassezia* cell pellets were washed three times with PBS and resuspended in 1 mL of TRIzol^TM^ (Thermo Fisher Scientific, Singapore). Cell lysis was performed by bead beating with 0.5 mm zirconia beads (BIOSPEC, Bartlesville, OK, USA) using a FastPrep-24^TM^ beat beater for 30 s at 6.0 m/s three times. The supernatant was combined 1:1 with 100% ethanol and subsequently processed with the Direct-zol^TM^ RNA MiniPrep kit (Zymo Research, Singapore), as per the manufacturer’s instructions. Additional TURBO^TM^ DNase (Thermo Fisher Scientific, Singapore) treatment of the samples was carried out to eliminate DNA contaminants. RNA quality was assessed with absorbance measurements (NanoDrop^TM^ One Spectrophotometer; Thermo Fisher Scientific, Singapore).

### 2.6. Identification of CBS 7982 M. furfur OPT1, FLR1 and CAF5 Homologs

To identify and validate the relevant gene sequences for OPT1, FLR1 and CAF5 in *M.* *furfur* (CBS 7982), OTUs blast-matched to the relevant *Malassezia* reference sequences were blasted into the CBS 7982 genome (BioProject ID: 286710) to find the most closely matched gene regions. Full-length exons were defined based on their start and start codon positions using SnapGene software version 8.2 (GSL Biotech LLC, San Diego, CA, USA). Gene sequences were translated into protein sequences using the ExPASy translate tool (https://web.expasy.org/translate/, assessed on 19 January 2020) and analyzed using multiple-sequence alignment (http://multalin.toulouse.inra.fr/multalin/multalin.html, assessed on 19 January 2020) to confirm protein homology to the identified protein families [25,26]. The chromosome position and gene regions of the respective genes are shown in Table 1 below. These gene sequences were used for subsequent primer design and gene analysis of the respective genes. For CBS 14141, the reference genome ASM993813v1 (GenBank ID: GCA_009938135.1) was used.

To elucidate the functional and physical interaction networks of the respective proteins, the STRING database (http://string-db.org/, assessed on 29 October 2025) was used to perform network analysis. Due to the absence of well-annotated *Malassezia* data in the STRING database, *S. cerevisiae* was used as the reference organism for evaluating the protein interaction networks for PDR10, OPT1 and FLR1. *S. pombe* was used as the reference organism for the CAF5 protein as it is not found in *S. cerevisiae*.

### 2.7. Gene Expression Analysis

Primers were designed and used for the amplification of the relevant gene regions listed in Table 1 below, and 100–200 bp primer amplicons were validated for their target regions by sanger sequencing. For quantification of gene expression, reverse transcription quantitative PCR analysis was performed on 10 ng of RNA per reaction in triplicate using the GoTaq^®^ 1-Step RT-qPCR system (Promega, Singapore) with the respective gene-specific primers using *Malassezia* actin *ACT1* as the housekeeping gene. Relative gene expression was analyzed using the qene module as described by Muller et al., 2002 [27]. Primer standard curves, efficiency and melt curves are available in Supplementary Figure S1.

## 3. Results

### 3.1. Prolonged Clotrimazole and Ketoconazole Exposure Drives Broad-Spectrum Antifungal Cross-Resistance

**Figure 1:**
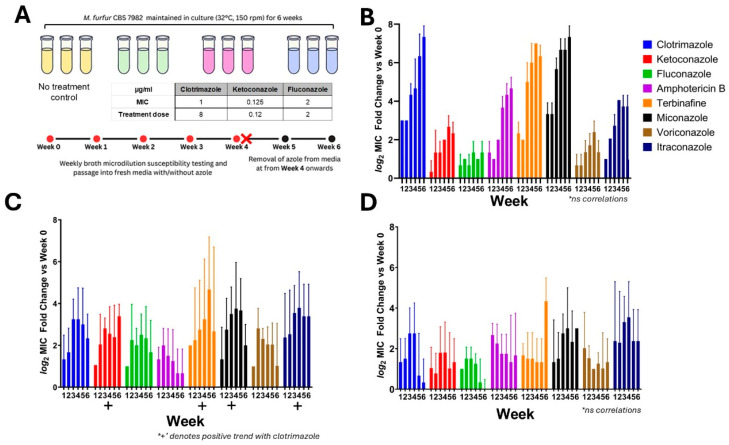
(**A**) Schematic of the experimental setup and bar graphs for treatment groups of ketoconazole, (**B**) clotrimazole (**C**), and fluconazole (**D**) over 6 weeks as measured by log2 MIC fold change over the Week 0 MIC (right). A one-tailed P test was performed using Spearman’s correlation with 95% confidence intervals. ‘+’ denotes a positive trend with clotrimazole. For comparisons within the same antifungal group, statistical significance was determined using a two-way ANOVA followed by Tukey’s post hoc test for multiple comparisons. Significant differences were defined as *p* < 0.05 (refer to Supplementary Tables S2 and S3 for values).

We previously observed that sustained sublethal clotrimazole exposure (two-fold higher than MIC) induced a transient increase in MIC values, which peaked at Week 4. Notably, this phenotype was reversible, as MICs declined toward baseline levels following the withdrawal of antifungal selection during Weeks 5 and 6 [14]. To determine whether this trend was reproducible for other azoles, we treated *M. furfur* CBS 7982 with MIC-level concentrations of ketoconazole and fluconazole for four weeks (Figure 1A) and monitored their antifungal susceptibility profiles before and after treatment (Figure 1B–D). The strain was unable to grow stably in above-MIC doses of ketoconazole (>0.125 µg/mL) and fluconazole (>2 µg/mL) for extended periods (>48 h).

Treatment with ketoconazole caused statistically significant increases in MIC values for several antifungals (clotrimazole, miconazole, and terbinafine), and the MICs did not recover on removal of ketoconazole from the media at Week 5 (Figure 1B, Supplementary Table S2). There was a consistent positive trend (Spearman’s, * *p* < 0.05, Supplementary Table S3) between the MIC trend for clotrimazole treatment compared to ketoconazole, terbinafine, miconazole and itraconazole (Figure 1C). However, this was limited by the small sample size (*n* = 4). Fluconazole treatment induced a nominal increase in MICs across the azole panel; however, these shifts did not reach statistical significance, suggesting a less robust selective pressure than that observed with clotrimazole or ketoconazole (Figure 1D).

### 3.2. Identification and Validation of OPT1, FLR1 and CAF5 Gene Regions

Three putative transporter genes, OPT1, FLR1 and CAF5, were identified based on previous RNA sequencing analysis [14]. These were OTUs mapping to putative gene regions coding for ‘Similar to *S. cerevisiae* protein OPT1 (Proton-coupled oligopeptide transporter of the plasma membrane, ATCC 42132)’, ‘Similar to *S.cerevisiae* protein FLR1 (Plasma membrane transporter of the major facilitator superfamily) [*Malassezia sympodialis* ATCC 42132]’ and CAF5 (Caffeine resistance protein 5, *Malassezia restricta* CBS 7877) (Table 2) [14]. These sequences were mapped to their relevant chromosomes in the *M. furfur* strain CBS 7982 (Table 2). FLR1 and CAF5 were mapped to single exons on chromosomes V and VII respectively. OPT1 was mapped to two adjacent homologs on ORF1 and ORF2 which we will call OPT1_01 and OPT_02 respectively. The two sequences share 90% sequence identity.

### 3.3. FLR1 and OPT1_02 Gene Expression Increases on Azole Exposure

**Figure 2:**
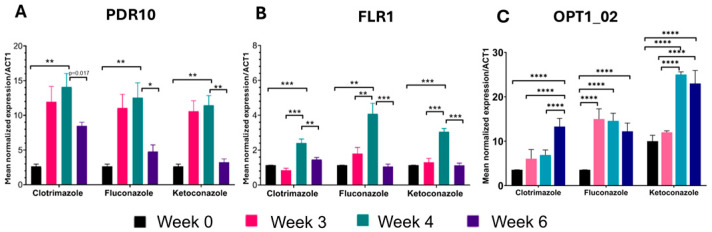
(**A**) Gene expression of PDR10, (**B**) FLR1 and (**C**) OPT1_02 under clotrimazole, fluconazole and ketoconazole treatment, respectively, as normalized to ACT1. Values shown represent mean ± SD. One-way ANOVA was used followed by Dunnett’s test (* *p* < 0.05, ** *p* < 0.01, *** *p* < 0.001, **** *p* < 0.0001). Data is representative of three biological repeats.

Consistent with previous reports of clotrimazole-induced upregulation [14], we observed that fluconazole and ketoconazole also triggered significant increases in *PDR10* expression over four weeks (Figure 2, ** *p* < 0.01). The expression kinetics followed a uniform pattern across the azole panel: transcript levels correlated positively with exposure duration but reverted toward baseline following the removal of antifungal pressure.

**Figure 3:**
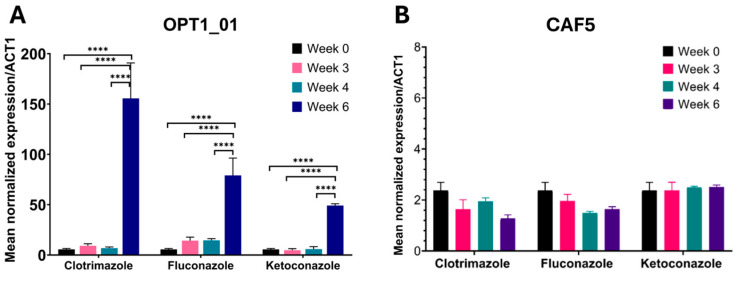
(**A**) Gene expression of OPT1_01 and (**B**) CAF5 under clotrimazole, fluconazole and ketoconazole treatment, respectively, as normalized to ACT1. Values shown represent mean ± SD. One-way ANOVA was used followed by Dunnett’s test (**** *p* < 0.001). Data is representative of three biological repeats. No statistically significant conditions (ns) were observed for all comparisons (**B**).

OPT1_02 expression increased with increased duration of exposure to azoles from Week 0 to Week 4 and was observed to have decreased for fluconazole and ketoconazole treatment by Week 6 (antifungals withdrawn from Week 5 onwards). OPT1_02 remained high even after removal of clotrimazole at Week 6. Ketoconazole induced the highest level of OPT1_02 expression from Week 0 to Week 3 (Figure 3, *p* < 0.001). FLR1 expression peaked on Week 4 for all azole treatment groups and decreased after the removal of drug treatment in Week 6 (Figure 3B).

### 3.4. OPT1_01 and CAF5 Gene Expression Is Not Affected by Azole Treatment

There was no significant increase in OPT_01 expression with exposure to the azoles on Week 3 and Week 4 compared to Week 0 (Figure 3). Expression levels increased significantly on Week 6 which corresponds to the timepoint after which azole treatment was removed. No significant changes in CAF5 expression were observed over the course of the different azole treatments.

To provide a foundational framework for these observations, we expanded our analysis through orthology-based comparisons with well-characterized fungal models like *S. cerevisiae* and *S. pombe* (Supplementary Figure S3). To understand the functional relationship between these four genes, we probed the STRING network database using *S. cerevisiae* as the reference organism for PDR10, FLR1 and OPT1. FLR1 was linked to PDR10 and its homolog, SNQ2, as a secondary node, suggesting that its increase in expression is linked to PDR10’s activity in the PDR network. OPT1 and CAF5 occupied nodes in separate networks, and neither PDR10 nor FLR1 were observed in any secondary nodes, suggesting that these proteins function independently.

### 3.5. MIC Values for the PDR10∆ Knockouts Strain Do Not Increase Under Antifungal Stress

**Figure 4:**
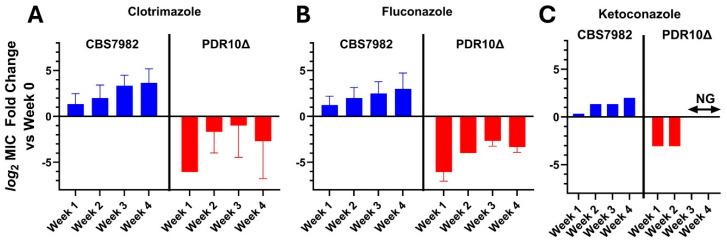
Log2 MIC fold change versus Week 0 MIC for strains CBS 7982 and PDR10∆ over four weeks of (**A**) clotrimazole, (**B**) fluconazole or (**C**) ketoconazole treatment. NG = no growth.

The PDR10∆ knockout strain was also subjected to the same extended antifungal treatment with clotrimazole, ketoconazole and fluconazole. However, no significant increase in MIC values was observed in all treatments (Figure 4). The PDR10∆ strain did not survive ketoconazole treatment beyond two weeks (Figure 4C). This suggests that PDR10 is a key mediator of reduced susceptibility to azoles, and knockout of the PDR10 transporter prevents them from acquiring additional resistance. However, their ability to tolerate low doses of clotrimazole, fluconazole and ketoconazole suggests that they still possess other means of drug efflux (e.g., FLR1 or OPT1) which may have implications on their susceptibility to other classes of antifungals.

To measure the activity of multidrug-resistant efflux pumps after the cells had been treated with the respective azoles, we used the rhodamine 6G fluorescent dye as an indicator of efflux pump activity. We did not observe any appreciable increase in rhodamine 6G efflux after treatment (Supplementary Figure S2). The PDR10∆ knockout strain showed elevated levels of efflux compared to CBS 7982 strains (treated and untreated). However, this is likely an intrinsic phenotype of the strain (its parent strain, CBS 14141, also has elevated rhodamine 6G levels) [14] rather than a result of the drug treatment. Consequently, some of these preliminary findings remain exploratory and inconclusive, requiring further investigation to fully elucidate the underlying mechanisms.

## 4. Discussion

The results of this study highlight a complex network of efflux machinery in *M. furfur* that underpins its ability to adapt to sustained antifungal pressure. We identified three transporter genes, PDR10, FLR1 and OPT1_01, to be significantly upregulated following extended clotrimazole exposure. While OPT1, a member of the oligopeptide transporter family, is traditionally associated with nutrient acquisition required for growth [28], emerging evidence suggests that certain members of this family may also facilitate drug resistance under specific stress conditions [29]. This functional flexibility is mirrored by FLR1, which serves as a primary drug transporter in the ascospores of *S. cerevisiae*, whereas Pdr5p remains the dominant active transporter in proliferating cells [30]. These observations suggest that specific ABC and MFS transporters play dominant roles depending on the duration and nature of drug exposure.

The presence of highly similar adjacent homologs in the *Malassezia* genome, such as SNQ2/PDR10 and OPT1_01/OPT1_02, further suggests an evolutionary mechanism designed to maximize environmental plasticity. Our data indicate that the expression of only one homolog may be required to generate acquired resistance, a phenomenon of partial redundancy and functional divergence that has been previously documented in other conserved gene duplicates, such as those in Arabidopsis thaliana [31]. OPT1 is part of the Oligopeptide Transporter Family (OPT1) which is responsible for the transport of essential biological molecules such as amino acids and peptides. CAF5 is most well characterized in *S. pombe* (no equivalent homolog in *S. cerevisiae*) and is involved in the Pap1 pathway which regulates oxidative stress. CAF5∆ have been demonstrated to be more susceptible to wild-type strains [32], and CAF5 overexpression confers caffeine resistance [33]. By identifying key “hub” proteins and their putative roles in the drug stress response, we hope to transition from preliminary observations to a more robust biological context that can explain the identified transporter interactions.

Our findings on the ability of single azole treatment regimens to elicit cross-resistance to other azoles are not surprising as imidazole and triazole drug classes are known to target the fungal enzyme, cytochrome P450 51A1. The extent of cross-resistance we are seeing is likely to vary with the specificity of specific azoles. Ketoconazole exhibits broad CYP51 inhibition that is ~11-fold higher [34] against fungal *CYP51* (IC_50_ ≈ 0.4 μM) compared to human CYP51A1 (IC_50_ ≈ 4.5 μM) [35], whereas fluconazole demonstrates >100-fold selectivity for fungal CYP51 over human orthologs due to poor binding affinity for the human active site. This would explain why ketoconazole treatment is able to elicit more cross-resistance to other azoles compared to fluconazole.

Despite these insights, several technical and experimental limitations must be acknowledged. Our efflux measurements rely on the rhodamine 6G assay, which require cells to be in a metabolically active log-phase. Because cells under extended antifungal stress may exhibit reduced metabolic activity, these assays may not actually capture the true efflux pump activity. Additionally, rhodamine 6G is primarily an ABC transporter substrate which may not accurately reflect the activity of MFS transporters. Given the dynamic changes in transporter gene expression over time, data collection from all time points and the identification of additional well-validated stable housekeeping genes in *Malassezia* would facilitate more accurate quantification of gene expression.

Alternative mechanisms independent of drug transporters have been characterized in other fungal models. In *Candida albicans*, reversible antifungal tolerance to ketoconazole has been linked to chromosomal plasticity, specifically the trisomy of chromosome R, whereby stress-response regulators Hsp90 and calcineurin are essential for the development and maintenance of this tolerant phenotype [36]. While we have identified stable phenotypic shifts for *Malassezia*, long-term reversion testing and high-resolution genetic characterization at multiple time points would be required to evaluate similar mechanisms in *Malassezia*.

Ultimately, these findings have significant implications for antifungal stewardship. The observation that ketoconazole treatment can elicit robust, non-reverting cross-resistance is particularly concerning given its widespread availability over the counter. Our data suggest that the use of ketoconazole as a first-line therapy may inadvertently reduce the efficacy of alternative azoles, increasing the risk of multidrug-resistant fungal infections. To mitigate this risk, clinical strategies should prioritize measures that limit unnecessary or prolonged azole exposure, incorporate the rotation of antifungal classes, and utilize mechanism-informed detection to slow the emergence of resistant strains. These perspectives emphasize early, mechanism-informed detection of resistance and individualized treatment plans to slow the emergence and spread of multidrug resistant fungal strains.

## Figures and Tables

**Table 1:** Primer sequences for target genes.

Gene ID	Forward (5′-3′)	Reverse (5′-3′)
ACT1	CCCGCTGAACCCSAAGGC	ACACCGTCACCCGAGTC
PDR10	ATGTTCCTGGCGTACTACCG	GACAGCACGAGCGACATAAA
FLR1	CCTCGCTTCAAACAAGGAAG	GAACGATGTCCAACCAAACC
CAF5	CAACGGTCGATACAATGTGC	ACGAGCGCTGATACGATTCT
OPT1_01	TTGAAGTTGAGCAAGCC	CACGATAGTACCCAGGA
OPT1_02	CGACGATTTCGGGTTACG	GTTGAACGAGAAATTGCC

**Table 2:** CBS 7982 *M. furfur* gene regions.

Gene Name	Gene ID	Chr.	Gene Regions	Homology (%)
Oligopeptide transporter 1	OPT1 OPT1_01 OPT1_02	I	17,014–19,499 19,961–22,414	*M. sympodialis* (64.53%) *S. cerevisiae* (37.12%) *S. cerevisiae* (37.01%)
Plasma membrane transporter of the major facilitator superfamily	FLR1	V	456,864–458,513	*M. sympodialis* (55.39%) *S. cerevisiae* (41.42%)
Caffeine resistance protein 5	CAF5	VII	163,117–164,200	*M. restricta* (72.76%) *S. pombe* (43.01%)

## Data Availability

The original contributions presented in this study are included in the article/Supplementary Materials. Further inquiries can be directed to the corresponding author.

## Supplementary Materials

The following supporting information can be downloaded at https://www.mdpi.com/article/10.3390/microorganisms14061315/s1, Figure S1: Primer standard curves were plotted using serial dilutions of *M. furfur* gDNA. Corresponding melt curves are shown in-set; Figure S2: Rhodamine 6G efflux was measured from cells treated with antifungals for 7 days, with readings taken at intervals of 5, 15 and 30 min in triplicate. Statistical significance was determined using a two-way ANOVA followed by Tukey’s post-hoc test for multiple comparisons. Significant differences were defined as *p* < 0.05; Figure S3: (A) STRING networks networks capturing the protein-protein interactions (PPI) between our gene of interest (in red circles)–SNQ2, PDR10 and FLR and (B) OPT1 based on known *S. cerevisiae* gene annotations and (c) CAF5 based on *S. pombe* gene annotations. Nodes in the network represent the protein and the links represent the interaction between the protein. Coloured nodes depict first shell of interactions; white nodes depict second shell of interactions; Table S1: MIC raw data tables (Excel spreadsheet); Table S2: Two-way ANOVA for clotrimazole, ketoconazole and fluconazole treatment groups (Excel spreadsheet); Table S3: Spearman’s Correlation, 95% CI.
