# Supplementary material for: Azole-Driven Cross-Resistance and Transporter Gene Expression in Malassezia Yeasts
Source: Microorganisms. 2026 Jun 12;14(6):1315. doi: 10.3390/microorganisms14061315 (PMC13303717; doi:10.3390/microorganisms14061315)
Supplement: Supplementary file 1 [file microorganisms-14-01315-s001.zip › Supplementary Material_Revised.pdf]

Supplementary Material

Azole-driven cross-resistance and transporter gene expression in *Malassezia* yeasts

Soo Ying Zhou[1,] Lee Shi Mun[1], Thomas L. Dawson, Jr[1,2,] Cheryl Leong[1#]

[1] A*STAR Skin Research Labs (A*SRL), Agency for Science, Technology and Research (A*STAR) & Skin Research Institute of Singapore (SRIS), 11 Mandalay Rd, #17-01, Singapore 308232, Republic of Singapore

[2]Center for Cell Death, Injury & Regeneration, Departments of Drug Discovery & Biomedical Sciences and Biochemistry & Molecular Biology, Medical University of South Carolina, Charleston, SC

Short title:

Azole-driven cross-resistance and transporter gene expression in *Malassezia* yeasts

#Address correspondence to:

Cheryl Leong

A*STAR Skin Research Labs

8A Biomedical Grove, #06-06,

Immunos, Singapore 138648

Singapore

Key words: antifungal, cross-resistance, azoles, *Malassezia, ABC transporters*

Supplementary Figures

Supplementary Figure S1. Primer standard curves, melt curves and efficiency

Supplementary Figure S2. Rhodamine 6G efflux

Supplementary Figure S3. Protein interaction networks as a framework for the elucidation of drug transporter function in *Malassezia*

Supplementary Tables

Supplementary Table S1. MIC raw data tables (Excel spreadsheet)

Supplementary Table S2. Two-way ANOVA for clotrimazole, ketoconazole and fluconazole treatment groups (Excel spreadsheet)

Supplementary Table S3. Spearman's Correlation, 95% CI

**Comparison (Weeks 1-4)**		**P value (one-tailed)**	**Significance**
Clotrimazole	Ketoconazole	0.0417	*
vs	Fluconazole	0.375	**ns**
	Amphotericin B	0.4583	**ns**
	Terbinafine	0.0417	*
	Miconazole	0.0417	*
	Voriconazole	0.4583	**ns**
	Itraconazole	0.0417	*

Supplementary Figure S1.

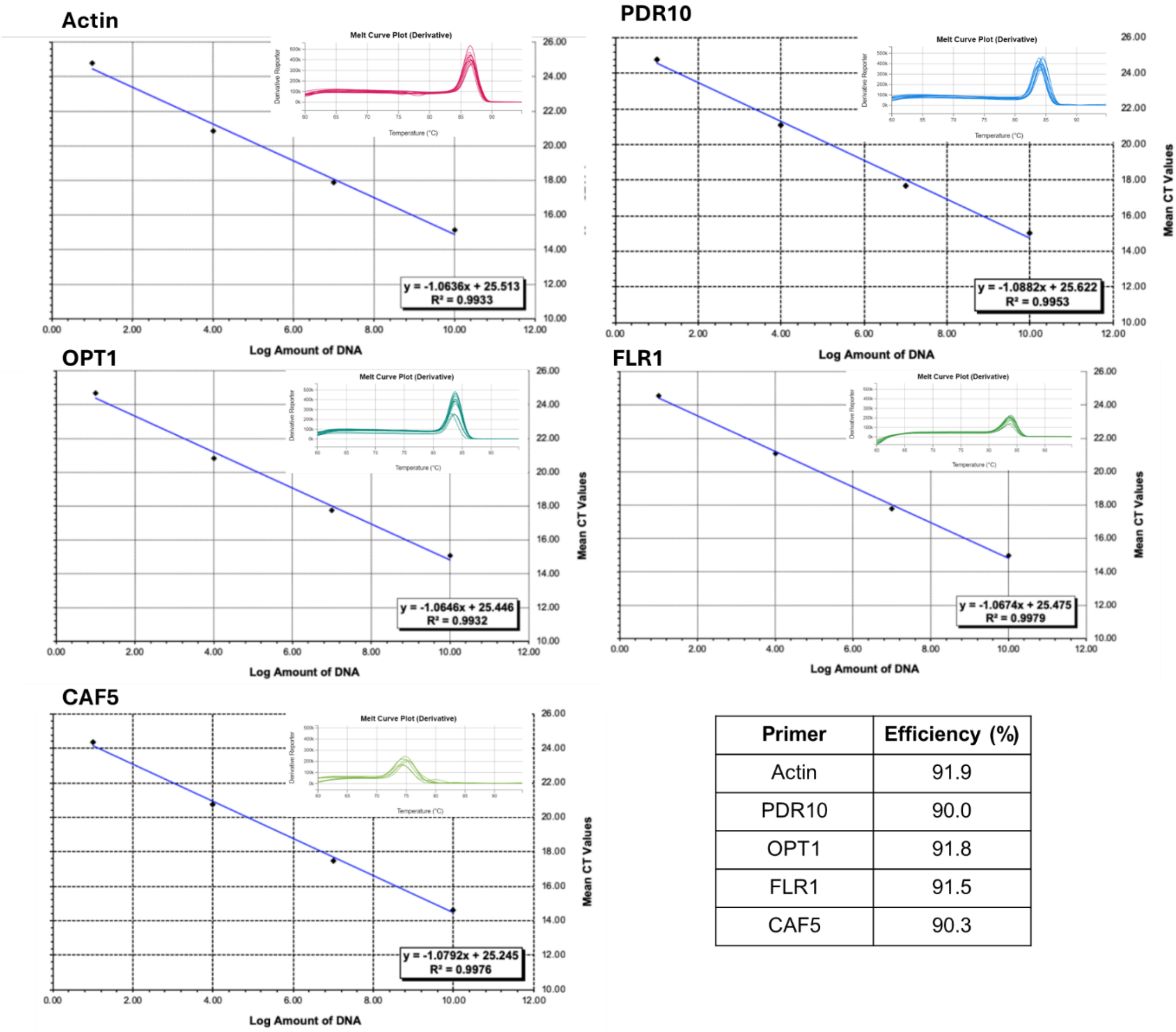

Primer	Efficiency (%)
Actin	91.9
PDR10	90.0
OPT1	91.8
FLR1	91.5
CAF5	90.3

Supplementary Figure S1. Primer standard curves were plotted using serial dilutions of *M. furfur* gDNA. Corresponding melt curves are shown in-set.

Supplementary Figure S2.

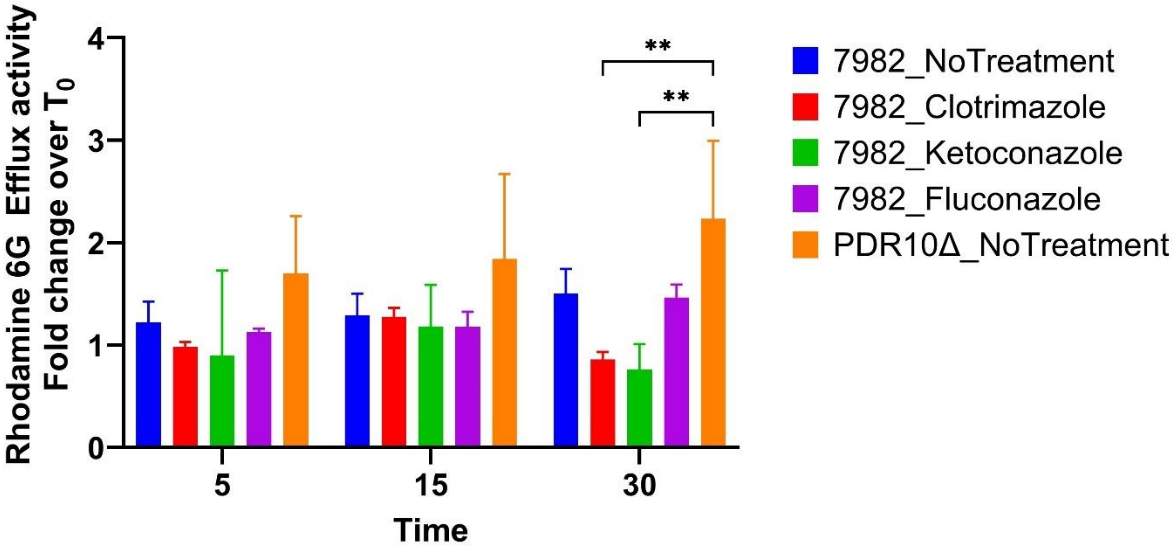

Supplementary Figure S2. Rhodamine 6G efflux was measured from cells treated with antifungals for 7 days, with readings taken at intervals of 5, 15 and 30 minutes in triplicate. Statistical significance was determined using a two-way ANOVA followed by Tukey's post-hoc test for multiple comparisons. Significant differences were defined as $p<0.05$.

Supplementary Figure S3.

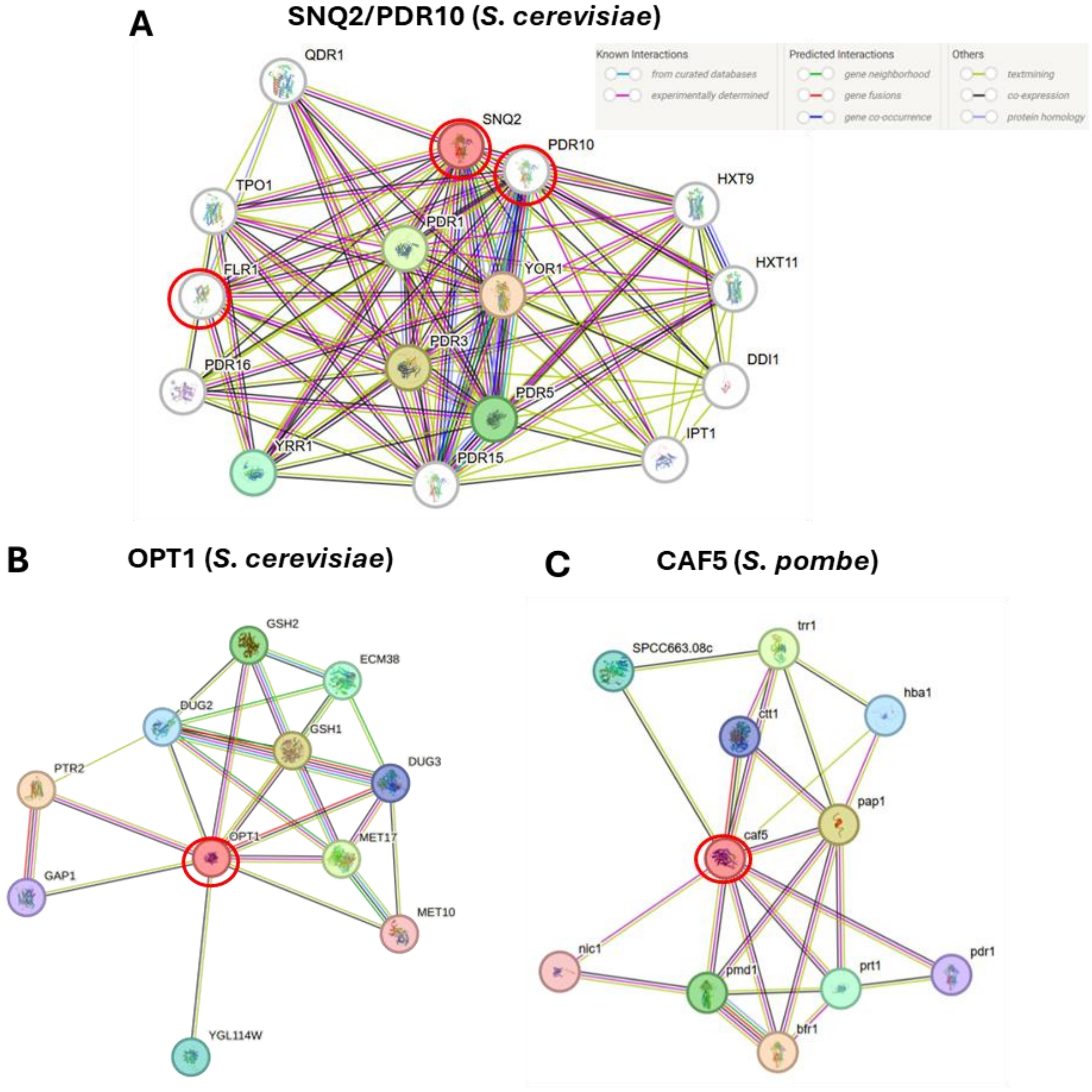

Supplementary Figure S3 (A) STRING networks networks capturing the protein-protein interactions (PPI) between our gene of interest (in red circles) – SNQ2, PDR10 and FLR and **(B)** OPT1 based on known *S. cerevisiae* gene annotations and (c) CAF5 based on *S. pombe* gene annotations. Nodes in the network represent the protein and the links represent the interaction between the protein. Coloured nodes depict first shell of interactions; white nodes depict second shell of interactions.
